# A comparative study of serum lipid contents in pre and post IFN-alpha treated acute hepatitis C patients

**DOI:** 10.1186/s12944-015-0119-x

**Published:** 2015-09-24

**Authors:** Sadia Qamar Arain, Farah Naz Talpur, Naseem Aslam Channa

**Affiliations:** National Centre of Excellence in Analytical Chemistry, University of Sindh, Jamshoro, 76080 Pakistan; Institute of Biochemistry University of Sindh, Jamshoro, Pakistan

**Keywords:** Hepatitis C virus, Alpha-2b interferon, Fatty acids, Cholesterol, GC-FID, Triacylglycerol, High density lipoprotein, Low density lipoprotein, Saturated fatty acid, Polyunsaturated fatty acid

## Abstract

**Background:**

To study the effect of Interferon (INF) alpha-2b therapy on the serum lipids and fatty acid (FA) level in pre and post treated hepatitis C (HCV) patients.

**Methods:**

Fifty samples were collected from pre and post treated patients along with age and gender matched controls. After separating serum, lipid contents were analyzed by microlab and gas chromatography.

**Results:**

The hepatitis C infection results in hypolipidemia with reduced level of triglyceride (113 mg/dl), high density lipoprotein (37.1 mg/dl), low density lipoprotein (74.3 mg/dl), cholesterol (149.9 mg/dl) that increase the infection resolution and after the IFN treatment, the lipid profile of the patients were increased. The myristic (2.8 g/100 g) and palmitic acids (26.6 g/100 g) were significantly higher while linoleic acid (20.94 g/100 g) was significantly lower in HCV patients. The higher oleic: stearic (1.4) and palmitoleic: palmitic acid (0.2) ratios were detected in HCV patients, showing enhanced stearoyl-CoA desaturase activity. The levels of serum saturated (44.9 g/100 g) and monounsaturated FA’s (26.98 g/100 g) were higher while polyunsaturated FA’s (25.9 g/100 g) were found lower in HCV patients in comparison of controls (40.1; 25.01; 33.44 g/100 g respectively). An inverse correlation was found HCV RNA viral load and PUFA (R^2^ = 0.4555). Elevated levels of serum saturated free FA (45.7 g/100 g) in HCV patients indicates stimulated lipoapoptosis.

**Conclusion:**

The present study conclude that serum PUFA level was lower in HCV patients, hence PUFA may provide synergistic antiviral effects when given as a food supplement during the INF based anti- HCV therapy.

## Introduction

The viruses of family *flaviviridae* cause the most widely spreading diseases across the world [1]. Among those diseases hepatitis C virus (HCV) is predominant one which belongs to genus *Hepacivirus* [2]. Worldwide, liver cancer is the second leading cause of cancer related death in men and the sixth in women. Due to hepatitis B virus (HBV) or HCV approximately 78 % of hepatocellular carcinoma (HCC) appears in patients which in turn accounts for 80 % of liver cancer cases [3].

In Pakistan, hepatitis B and C infection rate is increasing gradually due to unavailability of appropriate health facilities or poor socio-economical status and less public awareness about the transmission of hepatitis B, C and human Immunodeficiency Virus [4]. Patients suffering with hepatitis C virus leads to 29 % of patients with chronic liver disease and 8 % of patients with hepatocellular carcinoma in Pakistan [5, 6].

The liver plays a central role in lipid metabolism, and major metabolic processes take place at this level, involving the production, transportation and storage of lipoproteins, as well as catabolism of various lipids including excretion of cholesterol and phospholipids. An alteration in liver functions resulting from cellular injury leads to changes in the serum concentration of cholesterol and lipoproteins. An HCV virus leads to hepatic damage, which in turn relates to changes in alterations of the lipid metabolism [7].

Both fatty acids (FA) and cholesterol are playing major role in progression of HCV. Lipid storage in liver is leading to steatosis, which is caused by de novo lipogenesis, may contribute to HCV-induced damage by involvement of viral replication process. Sterol regulatory-element binding protein 1c and FA synthase enzyme is involved in lipogenic pathway and HCV is proposed to stimulate hepatic FA synthesis by up-regulation of these enzymes [8].

Progress seems to be happening rapidly in the Hepatitis C drug treatment arena. Approved by the U.S. Food and Drug Administration (FDA) in 2011, Incivek was apparently astounding new Hepatitis C drug. While it greatly improved the likelihood of beating the Hepatitis C virus in 2011, Incivek has already been discontinued effective October 2014. At the end of 2014 of effective interferon-free, hepatitis C treatments with Gilead’s Sovaldi, Gilead’s Harvoni and AbbVie’s Veikira Pak has been introduced. Although they may or may not also require the addition of ribavirin during the course of therapy, these effective, all oral medications come with a high price tag. However, interferon alfa-2b is FDA approved to treat chronic hepatitis C as well as hairy cell leukemia, malignant melanoma, condylomata acuminate and AIDS-related Kaposi’s sarcoma [9].

The standard interferon alpha -2b and interferon plus ribavirin therapies is to be considered the first line treatment for chronic hepatitis C patients. These therapies have been used in Pakistan since 1998 till now [10].

Although Interferon (IFN) is the one of the proven efficacy in the treatment of hepatitis C, though its mechanisms of treatment failure are inadequately understood, earlier reports have proposed IFN-stimulated genes and the inability to develop effective anti-HCV immunity as possible explanations [11]. In liver diseases, especially in non-alcoholic steatohepatitis, the effect of impaired peroxisomal polyunsaturated fatty acid (PUFA) metabolism and non-enzymatic oxidation on FA constitution is associated with disease progression [12]. It has been reported that HCV core protein has effects on FA synthesis, and that fatty droplets in the liver are related to development of disease. However the relationship between serum FA and efficacy of IFN-based antiviral therapy against HCV remains controversial [13].

Thus the aim of current study is to determine the variations in serum lipid and FA due to interferon alpha-2b treatment in pre and post treated acute HCV patients in comparison of healthy controls.

## Material and methods

Acute HCV infection is defined as the inflammation resolves within 6 months. Acute HCV patients were diagnosed by center of diseases control (CDC) criteria; the patients do not have symptomatic non-specific symptoms that may include malaise, anorexia, and abdominal pain [14]. The patients came in Taluka hospital TandoAdam with these symptoms, their blood samples were subjected to ELISA and ALT test. Elevated ALT levels were found in HCV positive patients (Table 1). The results were further confirmed by PCR HCV RNA active replication (>10–50 reactive and <10 non-reactive). All patients signed a written consent and were enrolled in the study. The study was approved by ethnic committee Institute of Biochemistry University of Sindh, Jamshoro.

**Table 1:** Demographic characteristics of HCV patients and controls

Demographic characteristics	Controls	Patients
Mean age (years)	34.2 ± 3.8	34.1 ± 4.2
Mean body weight (Kg)	58.0 ± 8.2	56.6 ± 7.5
Body mass index (BMI)	22.4 ± 1.2	23.1 ± 1.5
ALT levels (IU)	26.5 ± 14.1	77.6 ± 33.4

The male and female patients (age 18–56 years) with acute hepatitis C (*n* =100) were enrolled before starting the antiviral therapy during February 2012 to December 2013 treated in Taluka hospital TandoAdam, district Sangher, Sindh Pakistan. The patients were treated by Ceron alpha (interferon alpha 2b, injection 3MIU). Diabetic, hypertensive and having hepatitis co-infection, those taking supplementation of antioxidants, vitamins and PUFA, having pregnancy or lactating patients were excluded from present study. Age and sex matched controls (*n* = 50) having normal ALT levels and negative HCV RNA (ELISA) were selected for comparison.

Five ml intravenous blood samples from pre and post treated (after 1 week of treatment) patients and controls were collected after 14 h over night fasting. Serum was separated and stored at – 40 °C until analyzed for lipid profile and fatty acids by microlab 300 and gas chromatograph 8700 (Perkin–Elmer Ltd). Lipid profile include total cholesterol (TC), triacylglycerols (TAG), total lipid (TL), low density lipoprotein - cholesterol (LDL-C), high density lipoprotein - cholesterol (HDL-C), by kit method (Merck, Germany) based on microlab measurements [15]. FA composition was analyzed by gas chromatography (GC). All solvents and reagents used during the study were of analytical grade.

FAs were analyzed as total fatty acid (TFA) and free fatty acid (FFA). The samples for TFA and FFA were prepared as per reported method [16].

2FA analysis was performed on a model 8700 Perkin–Elmer Ltd., (Buckinghamshire, England) fitted with nonbonded biscynopropylsiloxane stationary-phase, polar capillary column Rt-2560 (100 m × 0.25 mm) 0.2 μm film thickness (Supelco, PA, USA) and Flame ionization detector. Oxygen-free nitrogen was used as a carrier gas at a flow rate of 3.5 mL/min. The initial oven temperature was 120 °C at rate of 4 min which was raised to 220 °C held for 20 min. The injector and detector temperature were set at 260 °C and 270 °C, respectively. A sample volume of 2.0 μl was injected.

Peaks were identified by authentic standards supplied by Fluka Chemika (Buchs, Switzerland). The FA standards, includes myristic acid (C14:0), myristoleic acid (C14:1), palmitic acid (C16:0), palmitoleic acid (C16:1w9), α-linolenic acid (C18:3w3), stearic acid (C18:0), linoleic acid (C18:2w6), oleic acid, vaccenic acid (C18:1), eicosapentaenoic acid (EPA (C20:5w3), arachidonic acid (C20:4w6) arachidic acid (C20:0), docosahexaenoic acid (DHA (C22:6w3), docosenoic acid (C22:1), nervonic acid(C24:1) and eicosatrienoic acid (C-20:3). The FA composition was reported as a relative percentage of the total peak area.

### Statistical analysis

The values are expressed as mean ± SD. For the association among the groups ANOVA was used with SPSS version 15 (SPSS Inc. Chicago, IL). *P* value is <0.05 considered statistically significant. Conditional logistic-regression analysis was performed using the SAS statistical software (version 9.1; SAS Institute, Inc., Cary, North Carolina). Odds ratios and 95 % confidence intervals (CI) were calculated to estimate the risk factor for quartiles as well as for continuous variables. Tests for trend were performed by using the means within each category in the logistic-regression model. Quartile cut points were determined by distribution of the fatty-acid levels among the referents, and the lowest quartile was used as the reference category.

## Results

Most commonly, acute hepatitis C infection is defined as the 6-month time period following acquisition of hepatitis C virus. The definition of acute hepatitis C is irrespective to whether the patient has clinical signs or symptoms of acute hepatitis. The rationale for choosing 6 months as the time period to define acute infection is based on evidence that most individuals who clear HCV will do so by 6 months. The CDC establish a gold standard for the laboratory diagnosis of acute HCV is anti- HCV seroconversion (negative anti-HCV before suspected exposure and positive anti-HCV following potential exposure), combined with a positive HCV RNA test and elevated ALT.

One hundred acute HCV patients consented and 30 patients were disqualified suffering from diabetes, malnutrition, hypertension, hyperthyroidism, renal failure, malignancy and immunoglobulin disorders. 20 patients were dropped out from the study, as they did not complete the 6 month treatment. Therefore only 50 patients completed the treatment and included in the present study. The median age of all patients was 34.1 years, BMI 23.1 and 50 % were female (Table 1). All patients were responders and completed the 6 month treatment and HCV RNA becomes non-reactive.

We found serum total lipids, total cholesterol, HDL-C, LDL-C, TAG in HCV pretreated patients were decreased as compared to controls. Whereas, after the interferon therapy lipid contents were increased and got back to normal ranges. The significant difference was observed in HDL-C and LDL-C and high HDL/LDL ratio shows coronary risk among the HCV patients as compared to controls (Table 2).

**Table 2:** Comparison of lipid profile of Control subjects with pre and post treated HCV patients

Lipid profile	Controls	HCV pretreatment patients	HCV post treatment patients
Cholesterol mg/dl	170.1 ± 9.7	149.9 ± 49.0	169.5 ± 18.8
LDL mg/dl	102.8 ± 5.2^a^	74.3 ± 35.9^b^	97.5 ± 2.9^c^
HDL mg/dl	55.5 ± 7.8^a^	37.1 ± 11.2^b^	45.7 ± 8.9^c^
Triglyceride mg/dl	126.5 ± 12.1	113.1 ± 54.1	123 ± 19.7
Total lipid mg/dl	543.8 ± 34.2	491.7 ± 161.0	533.2 ± 43.9
Coronary risk (HDL/LDL Ratio)	0.5 ± 0.07	0.6 ± 0.3	0.5 ± 0.1

Values represent mean ± Standard deviation of triplicates. Different alphabets on the same row indicate significant difference at *p* <0.05

*LDL* Low density lipoprotein, *HDL* High density lipoprotein, *TAG* Triglyceride

The result of total FA composition of pre and post treated acute HCV patients with contrast of healthy controls reveals that myristic and palmitic acids were significantly higher while linoleic acid was significantly lower in acute HCV patients. Stearic and docosenoic acids were elevated in pretreated patients and reduced after therapy. The palmitoleic acid was increased in both pre and post patients. The myristoleic, oleic, α-linolenic, arachidonic and arachidic acid were increased after INF treatment. The nervonic acid and DHA were not detected in both categories while in post treated acute HCV patients eicosatrienoic acid was not determined in comparison to healthy subjects. The oleic: stearic and palmitoleic: palmitic acid ratios, which are considered as a marker for stearoyl-CoA desaturase (SCD = ∆9-desaturase) activity was found higher in acute HCV patients, While PUFA: SFA was lower in acute HCV patients as compared with control subjects (Table 3).

**Table 3:** Total Fatty acid profile of HCV patients (pre and post) in comparison of controls

Fatty acids	Controls	HCV pretreatment patients	HCV post treatment patients
C – 14:0	1.1 ± 3.2^a^	2.8 ± 1.7^b^	1.9 ± 0.7^c^
C - 16 : 0	23.9 ± 5.3^a^	26.6 ± 3.8^b^	27.8 ± 2.3^c^
C - 18 : 0	14.7 ± 5.4	15.4 ± 5.12	12.3 ± 3.1
C - 20 : 0	0.4 ± 1.1	0.3 ± 0.9	0.4 ± 0.8
C - 14 : 1	0.5 ± 0.9	0.3 ± 2.2	0.4 ± 0.4
C - 16 : 1	2.6 ± 2.2	3.2 ± 1.4	3.8 ± 1.1
C - 18 : 1	19.8 ± 4.6	19.6 ± 3.6	20.9 ± 1.8
C - 22 : 1	1.9 ± 1.4	2.7 ± 1.4	2.3 ± 0.8
C – 24 : 1	0.3 ± 0.7	ND	ND
C - 18 : 2	25.0 ± 5.4^a^	20.9 ± 5.8^b^	21.6 ± 4.1^c^
C - 18 : 3	0.8 ± 1.3	0.3 ± 1.0	0.7 ± 0.9
C - 20 : 4	5.9 ± 2.1	5.1 ± 1.6	5.3 ± 0.8
C - 20 : 5	0.9 ± 1.7	0.6 ± 1.3	0.5 ± 0.9
C - 20 : 3	0.4 ± 0.6	0.2 ± 0.9	ND
C - 22 : 6	0.39 ± 0.7	ND	ND
C-18:1: C-18:0	1.3	1.3	1.7
C-16:1: C-16:0	0.1	0.2	0.3
PUFA: SFA	1.0	0.6	0.8

Values represent mean ± Standard deviation of triplicates. Different alphabets on the same row indicate significant difference at *p* <0.05. myristic acid (C14:0), myristoleic acid (C14:1), palmitic acid (C16:0), palmitoleic acid (C16:1), stearic acid (C18:0), oleic acid (C18:1), linoleic acid (C18:2), α-linolenic acid (C18:3), arachidic acid (C20:0), eicosatrienoic acid (C-20:3), arachidonic acid (C20:4), eicosapentaenoic acid (EPA (C20:5), docosenoic acid (C22:1), docosahexaenoic acid (DHA (C22:6), nervonic acid(C24:1), not detected (ND)

The serum free FA profile of acute HCV patient’s, shows significantly higher palmitic and stearic acid in pre-treated patients, which was reduced after therapy. The myristoleic, linoleic, arachidonic and EPA were significantly lower in acute HCV patients and increased after INF treatment. The EPA was not detected in post patients. The myristic, arachidic, palmitoleic acid were elevated in both pre and post-acute HCV patients. The oleic, α-linolenic, eicosatrienoic acid were reduced in acute HCV patients and the level of these FAs was enhanced after the treatment (Table 4).

**Table 4:** Free fatty acid composition of HCV patients (pre and post) in comparison of controls

Free fatty acids	Controls	HCV pretreatment patients	HCV post treatment patients
C – 14:0	1.5 ± 0.6	2.0 ± 1.1	2.0 ± 0.5
C - 16 : 0	23.8 ± 3.6^a^	28.6 ± 5.9^b^	25.8 ± 2.5^c^
C - 18 : 0	10.8 ± 4.1^a^	14.4 ± 5.4^b^	10.2 ± 1.7^a^
C - 20 : 0	0.5 ± 0.9	0.6 ± 2.1	1.6 ± 1.1
C - 14 : 1	0.1 ± 0.3^a^	ND^b^	0.2 ± 0.4^c^
C - 16 : 1	2.8 ± 1.4	3.5 ± 1.5	3.8 ± 1.2
C - 18 : 1	20.7 ± 2.9	19.9 ± 3.9	21.6 ± 2.1
C - 22 : 1	2.2 ± 1.3	2.2 ± 1.3	2.8 ± 1.0
C - 18 : 2	29.9 ± 4.6^a^	21.2 ± 4.6^b^	23.8 ± 5.6^c^
C - 18 : 3	0.4 ± 1.1	0.2 ± 0.6	0.4 ± 0.7
C - 20 : 4	6.1 ± 1.6^a^	4.4 ± 1.4^b^	5.3 ± 0.9^c^
C - 20 : 5	1.0 ± 1.6^a^	0.4 ± 0.9^b^	ND^c^
C - 20 : 3	0.2 ± 0.7	0.1 ± 0.5	0.5 ± 1.3

Values represent mean ± Standard deviation of triplicates. Different alphabets on the same row indicate significant difference at *p* < 0.05

Myristic acid (C14:0), myristoleic acid (C14:1), palmitic acid (C16:0), palmitoleic acid (C16:1), stearic acid (C18:0), oleic acid (C18:1), linoleic acid (C18:2), α-linolenic acid (C18:3), arachidic acid (C20:0), eicosatrienoic acid (C-20:3), arachidonic acid (C20:4), eicosapentaenoic acid (EPA (C20:5), docosenoic acid (C22:1), docosahexaenoic acid (DHA (C22:6), nervonic acid(C24:1), not detected (ND)

**Fig. 1:**
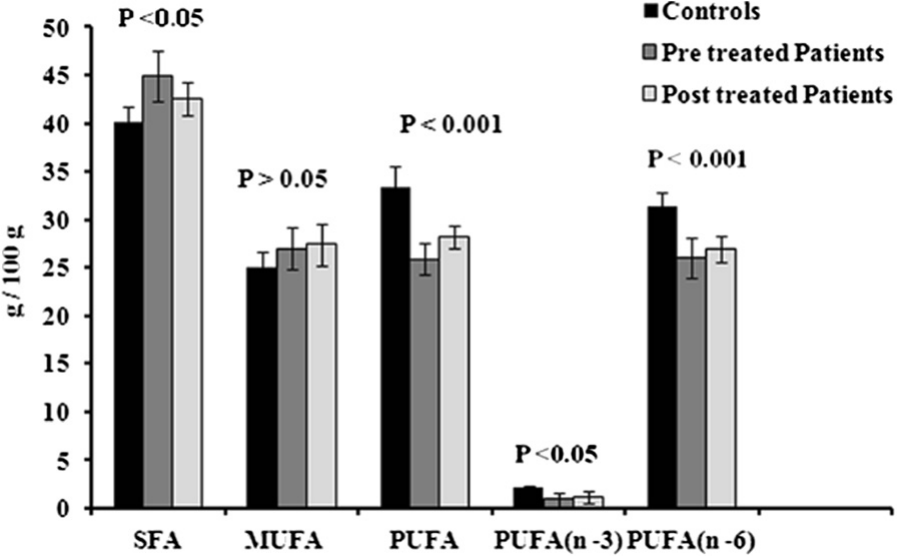
Comparison of SFA, MUFA, PUFA including n-3 and n-6 fatty acids in total FA composition of Controls and HCV patients (pre and post)

The total SFA and MUFA were higher while PUFA (n – 3 and n – 6) contents were lower in acute HCV pre-treated patients in comparison of controls. After the INF therapy the PUFA concentration was improved in patients (Fig. 1) but was found lower than controls.

**Fig. 2:**
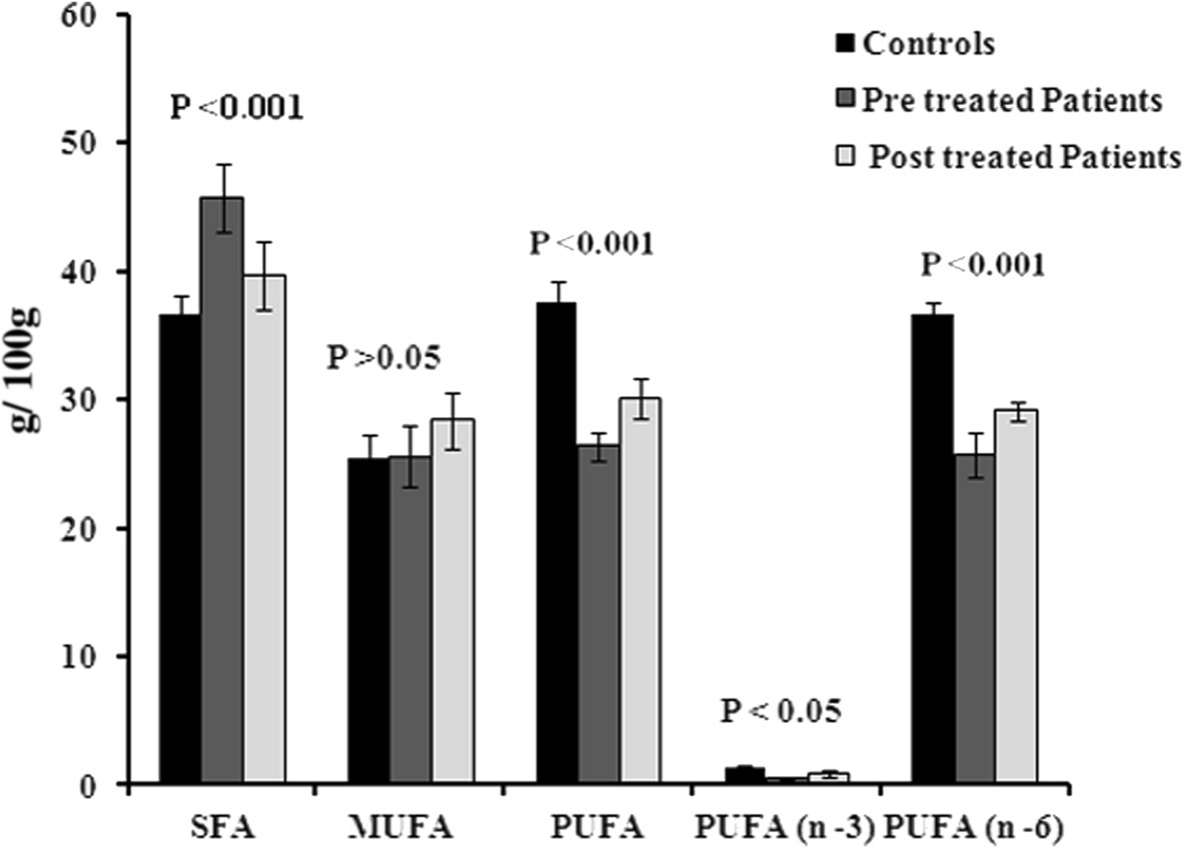
Comparison of Free SFA, MUFA, PUFA including n-3 and n-6 fatty acids in free FA composition of Controls and HCV patients (pre and post)

Figure 2, elucidates the free FA composition in acute HCV pre and post patients in contrast of controls. The saturated FFA was elevated, whereas the free MUFA and PUFA were lower in acute HCV patients in association of controls. After the INF therapy both MUFA, and PUFA were augmented and amount of SFA was abridged in acute HCV patients.

**Fig. 3:**
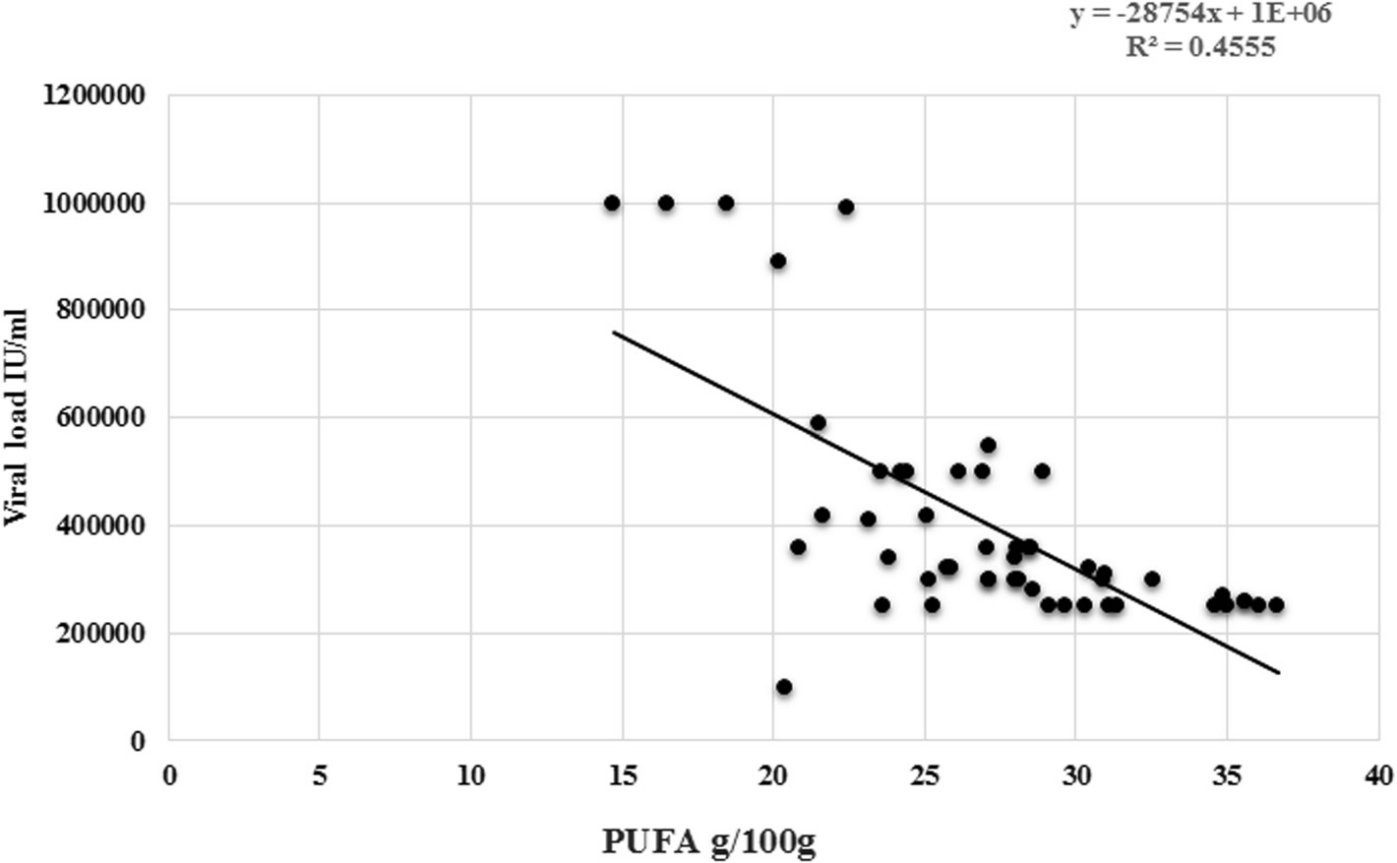
Correlation between HCV RNA viral load and PUFA among pre-treated HCV patients

The inverse correlations was found between viral load and PUFA levels with R^2^ 0.4555 as depicted in Fig. 3. Odds ratios were calculated (Table 5) for hepatitis C pretreated patients and controls by quartile of serum fatty acids. The significant association between serum fatty acids and hepatitis C virus was found when we compared the myristic acid odds ratio for the highest quartile with the lowest one 2.0 (95 % CI: 0.08, 97.8; *p* for trend = 0.0003), palmitic acid odds ratio was 2.6 (95 % CI: 0.4, 20.6; *p* for trend = 0.0002) and docosenoic acids odds ratio was 2.7 (95 % CI: 0.6, 12.2; *p* for trend = 0.001). Present study found an increased risk of hepatitis C virus replication associated positively with increasing levels of myristic, palmitic and docosenoic acids. On the contrary PUFA was inversely correlated with hepatitis C virus replication with odds ratio ≤0.9.

**Table 5:** Odd ratios for Hepatitis C patients and controls according to quintile of serum fatty acids

Fatty acids	Odds ratio (95 % confidence interval)					
	1st Quartile	2nd Quartile	3rd Quartile	4th Quartile	5th Quartile	*P* value
	Reference					
C – 14:0	1.00	1.8(0.4 – 9.3)	2.3(0.6 – 9.4)	2.6(0.5 – 13.4)	2.0 (0.04– 97.8)	0.0003
C - 16 : 0	1.00	1.8(0.3 – 13.5)	1.9(0.3 – 12.9)	3.4(0.6 – 20.3)	2.6(0.4 – 20.6)	0.0002
C - 18 : 0	1.00	0.3(0.06 – 1.7)	0.5(0.1 – 2.1)	0.8(0.2 – 3.3)	1.6(0.2 – 11.9)	0.21
C - 20 : 0	1.00	0.5(0.05 – 3.2)	0.9(0.02 – 35.3)	0.3(0.01 – 3.6)	1.9(0.1 – 53.4)	0.22
C - 14 : 1	1.00	0.8(0.02 – 35.8)	0.4(0.01 – 7.6)	0.4(0.01 – 7.6)	0.8(0.02 – 35.8)	0.27
C - 16 : 1	1.00	1.4(0.2 – 8.5)	2.1(0.4 – 11.1)	2.5(0.5 – 14.8)	1.4(0.2 – 8.7)	0.12
C - 18 : 1	1.00	2.7(0.4 – 18.8)	2.8(0.5 – 17.2)	1.1(0.2 – 7.9)	2.0(0.2 – 17.6)	0.411
C - 22 : 1	1.00	1.8(0.4 – 9.3)	2.5(0.6 – 11.1)	1.3(0.2 – 9.6)	2.7(0.6 – 12.2)	0.001
C - 18 : 2	1.00	0.5(0.08 – 2.3)	0.9(0.04 – 0.8)	0.2(0.04 – 0.9)	0.1(0.01 – 0.8)	0.002
C - 18 : 3	1.00	0.3(0.03 – 1.9)	0.1(0.004 - 0.8)	0.7(0.02 – 27.9)	0.4(0.01 – 5.5)	0.009
C - 20 : 4	1.00	0.8(0.1 - 4.6)	0.5(0.08 – 3.6)	0.5(0.08 – 3.3)	0.2(0.004 – 3.1)	0.008
C - 20 : 5	1.00	0.3(0.08 – 1.3)	0.2(0.008 – 1.9)	0.4(0.007 – 2.4)	0.7(0.07 – 8.0)	0.07
C - 20 : 3	1.00	0.1(0.005 – 1.0)	0.3(0.01 – 3.2)	0.8(0.02 – 31.2)	0.8(0.07 – 8.7)	0.17

## Discussion

We have reported the importance of lipid factors on IFN alpha – 2b therapy by evaluating lipid profile and FA composition of acute HCV patients before and after treatment. The low lipid profile was identified as contributing factor in acute HCV patients before treatment. Further analysis was performed to examine the relationship of FA levels to the response of IFN therapy. The IFN might alter the metabolism of lipids through receptors on hepatocytes, enterocytes and adipocytes which play major role in the serum lipids regulation. The most important fact is HCV replication was suppressed and hepatocytes may normalize their function because HCV has direct affect on hepatocyte lipid metabolic functions [17].

The present study suggests that elevated serum level of SFA and MUFA, while low level of PUFA could be a predictive factor for virological response to IFN-based therapy in acute HCV patients. The changes were reverted to base line after the treatment, significant increase in lipid profile was observed. The significance and mechanism of these changes is not clear, but it has been reported that HCV may alter expression profile of lipid metabolism–associated with genes [18]. Sterol regulatory element-binding proteins (SREBPs) are genes encoding enzymes involved in cholesterol and fatty acid biosynthesis, whereas SREBP-2 plays an important role in cholesterol biosynthesis, SREBP-1c is involved in fatty acid biosynthesis, which regulates the expression of its enzymes in the liver, e.g., fatty-acid synthase (FAS) and SCD [19].

The observed changes in FA composition in acute HCV patients indicate dysregulations of enzymatic conversion steps. The higher relative amount of MUFA and higher oleic: stearic and palmitoleic: palmitic acid ratios in HCV patients propose increased activity of SCD. It is rate-limiting enzyme involved in the synthesis of MUFA from SFA. As reported earlier that hepatic steatosis is developed by involvement of SDC, authors have shown that SCD1-deficient mice were resistant to diet-induced obesity and steatosis. The decreased lipogenesis and enhanced β-oxidation leading to the underlying mechanisms might involve regulation of transcription factors, like the SREBP-1 [20].

It has been well-known that infection with HCV virus leads to hepatic damage, which in turn relates to changes in alterations of the lipid metabolism. As evident from present study a consistent strong association exists between acute HCV infection and low levels of total cholesterol, TAG, LDL and HDL that increase the infection resolution. Corey *et al.*, [21] reported that chronic hepatitis C patients have lower cholesterol and LDL levels. This association persists when controlled for sex, race, and BMI, further strengthen this association by examining the change in lipid levels when hepatitis C is eradicated compared to patients who do not respond to treatment. Different mechanisms are involved, dependent on the stage of the liver disease and the metabolic state [22]. Moreover, there may be clinical implications for HCV-associated dyslipidemia [23]. Nutraceutical foods provide medical or health benefits for the prevention and treatment of diseases. The role of nutraceuticals and functional foods in dyslipidemia has been reported to act by reducing 7α-hydroxylase, increasing faecal excretion of cholesterol, decreasing 3-hydroxy-3-methylglutarylCoA reductase mRNA levels or reducing the secretion of very low density lipoprotein [24].

HCV infection can exert proatherogenic activities due to its direct action on vessel walls and/or via the chronic inflammatory process involving the liver [25]. The inflammatory condition related to liver infection could negatively influence the vascular wall performance and the cardiovascular system [26].

Several studies have shown that HCV core protein disrupts FA homeostasis. HCV core protein has been shown to significantly increase the proportion of oleic acid, but not palmitic acid, in the livers of patients with HCV infection. Irmisch *et al.* [27] compared FAs in serum of female patients with untreated chronic HCV infection with those treated with interferon-α and ribavirin and healthy controls. They showed that women who responded to treatment and healthy controls had significantly higher levels of eicosapentaenoic and arachidonic acid than did untreated HCV patients.

Present study found an increased risk of hepatitis C virus replication associated positively with increasing levels of myristic, palmitic and docosenoic acids. Previously, Kapadia *et al.*, [19] and Leu *et al.*, [28] have reported enhanced HCV replication due to increase in saturated FAs, including palmitic acid, whereas HCV replication was suppressed by increasing polyunsaturated FAs such as arachidonic acid *in vitro*.

Miyakes *et al.* [29] reported that the SFA may be responsible for impaired dendritic cells (DC) function. The inflammation and impaired antigen-specific function of DCs was due to palmitic acid in nonalcoholic fatty liver disease (NAFLD) in humans and mice. An additional explanation for the reduced effect of IFN-based therapy in patients is related with palmitic acid potency to develop ineffective anti-HCV immunity. These conditions are similar as found in present study with acute HCV infection. The impaired function of DC in acute HCV infection is possibly due to the reduced anti-HCV immunity by higher level of palmitic acid. Further studies are needed to identify the mechanisms which are involved in the effects of palmitic acid, including immunomodulatory effects.

In addition PUFA was found as protective factor for HCV replication as evident from odds ratio calculated for PUFA and viral load correlation. Huang *et al.* [30] showed that arachidonic acid has an ability to inhibit HCV replication by increasing lipid peroxidation, which results in decreased HCV RNA level. The physiologically relevant concentrations of arachidonic, DHA and EPA exert anti-HCV (hepatitis C virus) activities. The anti-HCV action of PUFAs is due to the formation of significant amounts of lipid peroxides. As observed in present study, an inverse correlation between HCV RNA and PUFA levels was found indicating that viral load increased with decrease in the PUFA concentrations. PUFAs have long been known to suppress hepatic lipogenesis, reduce hepatic triglyceride levels, and induce fatty-acid oxidation and degradation because previously it is demonstrated that HCV RNA replication requires fatty-acid synthesis [31]. The Sharookh *et al.*, [32] reported that PUFAs inhibit HCV RNA replication by a mechanism that is independent of their ability to inhibit lipogenic gene expression by antagonizing LXRα.

Moreover the chronic liver diseases had shown increased oxidative stress, due to hepatocellular iron accumulation and the direct effect of the HCV core protein. The reactive oxygen species including inflammatory cytokines such as tumor necrosis factor-α (TNF-α) and interleukin-1β produced during the immune response as activated phagocytes, which may cause oxidative stress [33, 34]. In present study lower EPA levels were observed in acute HCV pre and post treated patients. EPA has a high susceptibility to oxidation because it is a highly unsaturated fatty acid. The oxidative damage is prevented during treatment by antioxidant supplementation.

Arachidonic, DHA and EPA low levels were detected in acute HCV pre and post treated patients. The biosynthesis of various prostaglandins (PGs) and other eicosanoids are formed through FAs; as they are esterified in position 2 of glycerol and upon activation of phospholipases by physiologic or pathophysiologic stimuli; arachidonic and EPA are released and oxidized by cyclooxygenase and lipoxygenase. The synthesis of PGE2 from ω 6 PUFAs is accelerated and especially from arachidonic acid, which have an ability to suppress the production of type 1 cytokines [35]. The concentration of saturated FFA was higher in acute HCV pre and post treated patients. Malhi *et al.* [36] reported that cellular steatosis is caused by FFAs; which enhance expression of the apoptosis effectors TNF-α. The cytotoxicty of saturated FFAs was found higher than monounsaturated, therefore palmitic and stearic acid exhibited greater cytotoxicity than oleic and palmitoleic acid FFAs. The formation of reactive oxygen species in the alternative lipid metabolic pathways, including ceramide synthesis, modulation of death receptor expression and direct activation of cellular proapoptotic machinery are putative mechanisms involved in lipoapoptosis.

## Conclusions

In acute HCV patients, before treatment elevated levels of palmitic and myristic acid were found in serum, which may enhance virus replication and inhibits the anti- HCV effects of IFN therapy. The elevated levels of serum saturated Free FA induces lipoapoptosis which may cause cellular steatosis. PUFA play numerous important roles in normal physiological conditions and progression of diseases. The present study concludes that serum PUFA level was lower in acute HCV patients including arachidonic, α-linolenic and linoleic acids, which are reported to reduce HCV RNA level. Hence PUFA supplement can provide coadjuvant antiviral effects in the INF therapy.

### Limitations of the study

In present study numbers of samples are not large enough, though ideally larger group of subjects are required to delineate the role of lipid synthesis across the HCV spectrum.

## Abbreviations

DC: Dendrite cell
FAs: Fatty acids
FAMEs: Fatty acid methyl esters
FAS: Fatty-acid synthase
FID: Flame ionization detector
GC: Gas chromatography
HCV: Hepatitis C virus
HDL: High density lipoprotein
INF: Interferon
LDL: Low density lipoprotein
MUFA: Monounsaturated fatty acid
NAFLD: Nonalcoholic fatty liver disease
PUFA: Poly unsaturated fatty acid
SFA: Saturated fatty acid
SCD: Stearoyl-CoA desaturase
SREBPs: Sterol regulatoryelement-binding proteins
TAG: Triacylglycerol
TNF: Tumor necrosis factor
